# Long-term health effects of antipyretic drug use in the ageing population: protocol for a systematic review

**DOI:** 10.12688/f1000research.27145.1

**Published:** 2020-10-30

**Authors:** Seema Mahesh, Esther van der Werf, Mahesh Mallappa, George Vithoulkas, Nai Ming Lai

**Affiliations:** 1 School of Medicine, Taylor's University, Subang Jaya, Malaysia; 2 Centre For Classical Homeopathy, Bangalore, India; 3 Louis Bolk Instituut, Bunnik, The Netherlands; 4 Postgraduate Doctors Training Institute, Cheboksary, Russian Federation; 5 International Academy of Classical Homeopathy, Alonissos, Greece

**Keywords:** antipyretic, long term, elderly, older adults, aged, ageing, health effects

## Abstract

**Background:** Fever is suppressed with drugs due to discomfort and risk of organ damage. However, there is some compelling evidence for the benefits of fever. The elderly are a special population in this regard as they have a blunted fever response. The benefit-harm balance of antipyretic use in this population is unclear.

This study aims to provide the synthesized best evidence regarding long-term health effects of antipyretic treatment in the elderly during infections, investigating the onset/worsening of common chronic diseases, for e.g., thyroid disorders, connective tissue diseases and chronic obstructive pulmonary disease/asthma.

**Methods:** A systematic review will be performed to establish the best evidence available regarding antipyretic treatment in the elderly, searching databases such as Medline, Embase and Cochrane CENTRAL from their inception till date for all types of studies. Studies that consider the drugs in analgesic role will be excluded. The search will be reported following the ‘Preferred Reporting Items for Systematic Reviews and Meta-Analyses’ (PRISMA) guidelines. Randomized control trials, quasi experimental studies, observational studies, case series and reports will be included. The primary outcome measure being onset/worsening of chronic inflammatory diseases. Other outcomes include relief of symptoms, length of hospital stay, patient satisfaction, mortality, blood/immune parameters indicative of morbidity and complications of the infection.

Risk of biases in randomized studies will be assessed through the Cochrane risk of bias tool. For other study types, appropriate tools such as CASP/QUIPS/Cochrane non-randomised studies tool will be used. Meta-analysis will be conducted on the Cochrane RevMan software and where pooling of data is not possible, a narrative synthesis will be performed. Overall certainty of evidence will be assessed through the GRADE approach.

**Discussion:** The study aims to provide evidence regarding benefit-harm balance of antipyretic use in the elderly population to inform clinical practice and future research.

**Systematic review registration:** PROSPERO CRD42020160854

## Introduction

The leading cause for mortality has steadily shifted from communicable to non-communicable diseases over the past few decades. There has been a 14% increase in mortality due to non-communicable diseases since 1980, indicating a change in the global health scenario ^1^. Currently, the global burden of disease estimate shows that 73.4% of global deaths were from non-communicable disease causes ^2^. This is especially true of the ageing population ^3, 4^.

Research has shown that one of the chief mechanisms in the development of non-communicable (chronic) diseases is chronic low-grade inflammation, which the body is not able to resolve completely ^5^. Acute inflammation, a defence mechanism, when it fails to resolve, seems to perpetuate as chronic inflammation ^5, 6^. Vithoulkas and Carlino ^7^ have posited that repeatedly suppressing acute inflammatory diseases/fevers, through antipyretic/anti-inflammatory/antibiotic drugs, may lead to lack of efficient acute inflammatory response, resulting in a low-grade subacute inflammation. This may eventually turn into chronic inflammation. The immune system then loses the ability to mount an efficient acute inflammatory response. Here we find a plausible explanation for the elderly being unable to raise a high temperature; the process of aging involving chronic inflammation ^8^. This process is probably much slower in those endowed with very healthy and long lives, preserving their efficient acute inflammatory response ^8^. Vithoulkas and Carlino ^7^ propose that among the causes for the change in the global health scenario, from being predominantly infectious to chronic inflammatory diseases, the way acute inflammatory diseases are being over treated is also one. Fever, a highly regulated and essential physiological response, serves as an immediate booster of immune surveillance during infections. The febrile range of temperature acts on the various cell types involved in the tightly orchestrated acute inflammatory response ^9^. Chronic inflammation, on the other hand, has been found to be perpetuated by failure to resolve acute inflammation ^10^.

The elderly are known to have a blunted febrile response ^11^, due to decreased production of endogenous pyrogens, immunosenescence and hypo nutritional status ^8, 12, 13^. Such a blunted fever response is known to be associated with increased mortality, indicating the importance of a robust febrile response in infections ^14, 15^.

We must, however, also consider that high fever is proven to be dangerous in those whose cardiorespiratory reserve is compromised – for example as may happen in ischemic stroke and cardiac arrest. In these people therapeutic hypothermia is practiced, showing that hyperthermia and even normothermia is detrimental at times ^16, 17^. Fever suppression has long been shown to increase pathogen activity in the body ^18^. Whereas onset of fever has been shown to be beneficial and in some terminal cases even increase survival possibilities in the host ^19^. Lack of fever and leukocytosis led to increased mortality in community acquired pneumonia in the elderly, and another study has demonstrated that introducing acetaminophen, the common drug for fever, did not help reduce the intensive care unit admission days in the elderly with infection ^20, 21^. On the other hand, there is evidence showing the benefit of reducing fever, such as averting febrile convulsion in children, which may result in brain injuries and neuropsychiatric alterations ^22, 23^ and also in the elderly where the extra metabolic cost and the danger of organic damage from fever exists due to debilitating co-morbidities ^22, 24, 25^. Thus, while there is logic behind suppressing fever as it can be detrimental, it is not entirely clear in what way the presence of fever is beneficial or in which situation harmful to the body ^26, 27^. Whether to treat or not treat fever, especially in adults, has remained a debate with no consistent recommendation based on sound evidence ^28^. In view of the widespread global use of antipyretics, it is imperative to evaluate the best current evidence on the benefit-harm balance of antipyretic use for fever suppression.

### Objectives

This systematic review aims to synthesize evidence on the effects of antipyretic drugs in fever suppression during infection in the elderly, especially in the long-term.

This review will not explore the use of antipyretics for acute pain or other acute inflammatory conditions in the absence of fever.

### Research question

What is the long-term effect of antipyretic treatment in the elderly with fever from infectious aetiology?

## Methods

### Study design

This study will be a systematic review and meta-analysis (where applicable), to assess the long-term effects of antipyretic fever treatment during infections in the elderly. The focus of the study will primarily be on the long-term health effects concerning the onset of worsening of chronic inflammatory diseases common in elderly populations, such as thyroid disorders, connective tissue disorders and asthma. Besides, other outcomes from antipyretic treatment such as effect on fever symptoms, mortality, length of hospital stay and patient satisfaction.

A protocol for this systematic review and meta-analysis is registered with PROSPERO (International Prospective Register for Systematic Reviews; CRD42020160854) and the PRISMA-P checklist is provided as *extended data* ^29^. Any changes made thereafter will be updated on the PROSPERO database and reported in the final paper.

### Study eligibility criteria

***Population*.** As per the World Health Organization (WHO) classification ^30^, people aged 60 years and above with fever during infection will be included (studies will be included if the age definition is according to the definition of WHO; studies where the sample population was at least 70% comprising of the elderly or providing a subgroup analysis for this age group will also be considered). The population will include both healthy elderly individuals and those with any disease diagnosis.

Studies involving elderly people who are on regular analgesics/antipyretics for their chronic complaints will be excluded from the review.

***Intervention*.** We will include any study that includes treatment with any form of antipyretic for fever during infections in the elderly. Though many antipyretics are also analgesics, the role sought here is that of antipyresis and the analgesic role will not be included. Studies that do not clarify the intended antipyretic/analgesic role will not be included

***Comparator*.** We will include any study that compares the antipyretic drugs to any other treatment, placebo or no treatment.

***Outcomes***

(a) Primary outcomes:

Long term health effects from antipyretic treatment. Long-term in this context implies effect that lasts for over 3 months from the episode of fever treatment. Onset of most common chronic immune mediated/inflammatory diseases in the elderly viz.,

i) Thyroid disorders: Autoimmune disorders of the thyroid gland – autoimmune thyroiditis; hyperthyroidism e.g., Grave’s disease [E05 category of International Classification of Diseases 10 (ICD 10)] ^31^ and hypothyroidism, e.g., Hashimoto’s thyroiditis (E06.3 category of ICD 10) ^31^;
ii) Connective tissue disorders: Disorders involving the musculoskeletal system as elaborated in ICD 10 classification categories M02.8 – M03, M05 – 07, M10 – 12.4, M15 – 19, M30 – 36 (except the juvenile forms) M43, M60.1, M60.8 – 61.1, M61.5 and M61.9 ^31^;
iii) Chronic obstructive pulmonary disease/asthma: disorders involving the conditions as under ICD 10 classification category J40 - J47 ^31^;

(b) Secondary outcomes:

The effect of antipyresis during infection on:

i) Fever suppression: We will extract data from the studies that provide effect on temperature and discomfort associated with it during infection with antipyretic drugs.
ii) Length of stay at hospital: The studies that measure the number of days of hospitalization during infections and the effect of antipyresis will be considered and this specific data will be extracted from them.
iii) Patient satisfaction: If studies assess level of satisfaction in patients from treatment with antipyretics against no such treatment or placebo (usually done on predetermined scales of measurement), we will extract this data.
iv) Mortality: The data from studies that measure the mortality score with antipyresis during infection and compare with no treatment or placebo will be recorded.
v) Effect on blood/immune parameters indicative of morbidity (platelet count, erythrocyte sedimentation rate, C-reactive protein (CRP)/high sensitivity CRP and liver enzymes): Many studies measure the difference in immune and blood parameters during infection with and without antipyretic treatment. This is especially relevant in infections which have a great impact on the blood parameters such as dengue. Such data will be assessed for this outcome.
vi) Effect on further complications of the infection: If evidence exists on the influence of antipyretic treatment on preventing or facilitating complications then such studies will be analysed for their data.

*Subgroup analysis:* since the population in consideration for this study does not discriminate the different states of health at the outset, a subgroup analysis of healthy elderly, those with common chronic diseases and those with severe immune impairment/frail elderly, will be made in each of the above category if such data may be delineated.

***Study types*.** Randomized controlled trials, quasi experimental studies, non-randomized control trials, before and after studies, observational studies (retrospective and prospective), case control studies, and case reports and series will all be included, and the data will be pooled if possible.

### Search strategy

Search strategies for the respective databases will be designed to identify and collate studies over different databases, viz. PubMed, EMBASE, Cumulative Index to Nursing and Allied Health Literature (CINAHL), PsycINFO, Elsevier ClinicalKey, ScienceDirect, Cochrane Library, Clinicaltrials.gov and the World Health Organization (WHO) and International Clinical Trials Registry Platform (ICTRP). They will be searched from the inception of their records till the time of final analysis for this study. The search will include all studies that have reported fever treatment in the elderly during infections with regard to any of the said outcomes, comparing with no treatment/placebo/any other treatment.

The search will be conducted using Medical Subject Headings (MeSH) for PubMed and Emtree and keywords for EMBASE and other keywords denoting a combination of intervention (antipyretic, paracetamol, acetaminophen, placebo etc.) and population (older adults, aged, ageing, elderly etc.) for other databases. We will search only published studies and no grey literature will be included. A search strategy drafted for MEDLINE search is included as *extended data* ^29^.

A restriction will be made to obtain human studies only, but no restriction will be laid on language. An attempt to understand the non-English articles will be made through the Google translate application and if unable to understand despite this, a native speaker will be sought for help. The articles that are extracted will be screened for relevant referenced papers for those that may not be available through database search. Full text screened articles that are excluded will be done so with explanation for the exclusion.

The process of screening will be depicted in a Preferred Reporting Items for Systematic Reviews and Meta-analysis (PRISMA) flowchart.

### Data collection

The data will be collected using special data extraction forms predesigned for each type of study. Data will be collected as per the following categories from each paper extracted from the search:

- Journal name; title; authors; year of publication; funding sources.
- Type of study; study setting; sampling size; control and interventions arms; type of infection; type of intervention; details of intervention delivery - dose, combination etc; blinding
- Mean age; range of age; gender distribution; inclusion and exclusion criteria; health status
- Outcomes assessed; follow-up period; participant attrition; statistical analysis population (Intention to treat/per protocol); statistical analysis; conclusions.

The relevant statistical comparisons and measures will be extracted from these studies for each of the outcomes.

*Specific exclusion criteria:* studies dealing with effect of the said drugs in the role of analgesics with elderly population taking them on a regular basis will be excluded. Studies that make no distinction on the role of the drug as analgesic or antipyretic will be excluded.

***Missing data*.** Study authors will be contacted for any missing data and a reminder will be sent. On failure of response from authors, we will continue with the analysis of available studies as they are. Any missing data will be recorded as such and the whole sample (intention to treat) will be considered for analysis.

### Data analysis

Two investigators will independently review the studies for their eligibility and will also review the data collected as described above. Any disagreement or discrepancies between them will be appealed to the third reviewer whose decision shall settle the disagreement. The data will be summarized in a table showing the number of studies and their respective demographic, intervention and outcome measures. Mean, median, range and standard deviation will be calculated for continuous measures.

### Risk of bias

For the included randomized control trials, The Cochrane Bias tool 2.0 ^32^ assesses results of a study for bias through five measuring domains: selection, performance, detection, attrition, reporting. The biases are ranked according to the level of risk they pose - low/high/unclear. The quality of evidence will have to be agreed upon by both investigators who will independently assess them; any disagreements will be resolved by the third reviewer.

For other study types, such as non-randomized studies, observational studies etc., tools such as the Cochrane tool for non-randomized studies, Critical Appraisal Skills Program (CASP) (Nadelson & Nadelson, 2014) and Quality in Prognostic Studies (QUIPS) (Hayden, van der Windt, Cartwright, Côté & Bombardier, 2013) tool will be used.

### Statistical analysis

We will perform meta-analysis, where applicable, using Review Manager 5.3 (RevMan 2014) ^33^, with a random-effect model, following the recommendations of Cochrane. Where possible, our primary data analyses will follow the intention-to-treat principle: namely, the original number of participants allocated to each study arm will be used as the denominator in subsequent analyses. We will synthesize, for the sake of meta-analysis, the study results in the form of risk ratios, risk differences, number of patients needed to be treated (Benefit), number of patients needed to be treated (Harm) and weighted mean differences with their respective 95% CIs, as detailed under measures of treatment effect.

Studies that cannot yield these data will be excluded from the meta-analysis, and a narrative synthesis will be carried out for such studies according to the narrative synthesis framework of Cochrane consumers and communication group ^34^

Initially, all antipyretics, regardless of types, routes and duration will be grouped together. This is in order to find the overall effect and the degree of consistency of the findings between studies. If sufficient data are available, subgroup analysis will be carried out with respect to type, dose and duration of antipyretics. For example, dosage may be assessed under subgroups of standard dose/low dose/high dose; the route may be assessed under parenteral/oral/rectal suppositories; types may be assessed under non-steroidal anti-inflammatory drugs/acetaminophen/salicylates/others.

The overall certainty of evidence will be assessed using the GRADE approach (Grading of Recommendation, Assessment, Development and Evaluation) ^35^ and it will be categorized as high, moderate, low or very low. The certainty will be rated by default as ‘high’ if randomized controlled trials are the basis of the evidence, with downgrade of the certainty of evidence upon the identification of at least a serious concern on the following aspects: risk of bias, inconsistency, indirectness, imprecision and publication bias. On the other hand, the default rating for non-randomized studies is ‘low’, with possibility of upgrading the certainty of evidence based on the consideration of large effects, dose-response relationship and the presence of plausible confounders to the observed effects

### Dissemination

The authors intend to publish the completed review in a peer reviewed journal.

## Study status

Initial search has been made and studies have been downloaded for further screening and extraction

## Discussion

The question here is whether the elderly benefit from antipyretic treatment in the long term or if it causes damage. While the physician decides based on the immediate requirement of relieving the patient, it will help to know the outcome of such practice, especially if it can be avoided in cases where such treatment may be dispensed with. Most antipyretics, such as acetaminophen, act on the cyclo-oxygenase pathways and inhibit prostaglandin-E2, thus inhibiting fever. The mechanism may inadvertently be causing other long term effects ^36^.

The authors are not aware of any other systematic review addressing this particular question.

This study also aims to highlight gaps in clinical research on the long-term effects of antipyretics for aged patients that is used during infections.

## Data availability

### Underlying data

No data is associated with this article.

### Extended data

Figshare: Extended data_SeemaMahesh, https://doi.org/10.6084/m9.figshare.13116458.v1 ^29^.

This project contains the following extended data:

- Draft search strategy for Medline.
- Data extraction forms templates for different types of studies

### Reporting guidelines

Figshare: PRISMA-P checklist for ‘Long-term health effects of antipyretic drug use in the ageing population: protocol for a systematic review’, https://doi.org/10.6084/m9.figshare.13116458.v1 ^29^.

Data are available under the terms of the Creative Commons Attribution 4.0 International license (CC-BY 4.0).
