# Supplementary material for: Long-term health effects of antipyretic drug use in the ageing population: protocol for a systematic review
Source: F1000Res. 2020 Oct 30;9:1288. [Version 1] doi: 10.12688/f1000research.27145.1 (PMC9051583; doi:10.12688/f1000research.27145.1)
Supplement: Peer review history

# Peer review: Reviewer response for version 1

**DOI:** 10.5256/f1000research.29987.r128427

**Published:** 2022-04-28

**Authors:** Atakan Yilmaz

**Affiliations:** 1 Department of Emergency Medicine, Pamukkale University Hospital, Pamukkale University of Medical Science, Kinikli, Denizli, Turkey

An interesting study to determine the long-term health effects of antipyretic use in the elderly population. In terms of clinical treatment strategies, there may be differences between the young population and the elderly population in many issues. The elderly population is a special group for which many issues need to be studied. I will be eagerly awaiting the results of the study.

Is the study design appropriate for the research question?

Yes

Is the rationale for, and objectives of, the study clearly described?

Yes

Are sufficient details of the methods provided to allow replication by others?

Yes

Are the datasets clearly presented in a useable and accessible format?

Not applicable

Reviewer Expertise:

Emergency Medicine

I confirm that I have read this submission and believe that I have an appropriate level of expertise to confirm that it is of an acceptable scientific standard.

# Response

**Published:** 2022-04-28

**Authors:** Seema Mahesh

**Affiliations:** Taylor's University, Malaysia

Thank you very much for the review. The revision is now complete and I am awaiting it's publication. I will update the link here once it is published.

 Dr Seema Mahesh

# Peer review: Reviewer response for version 1

**DOI:** 10.5256/f1000research.29987.r80627

**Published:** 2021-03-22

**Authors:** Sylwia Elżbieta Wrotek

**Affiliations:** 1 Department of Immunology, Faculty of Biological and Veterinary Sciences, Nicolaus Copernicus University, Torun, Poland

This protocol describes methods how to investigate the effect of antipyretic drugs on long-term health of elderly people. Until now the effect of antipyretics was a subject of a few meta-analyses. The novelty of this study is the selection of population of elderly people.

 The authors will search a variety of databases to collect relative information on antipyretic drugs used in elderly. The study methods are appropriate and authors are going to use best practice standards for systematic review. Many tools to assess the risk of bias will be involved. Meta-analysis will be conducted and the GRADE approach for evaluating the quality of evidence will be used. In general this proposal is well planned and this research will be interesting and important. In my opinion sufficient details are provided to allow the researchers to follow.

 This protocol however can be improved and especially the background should be developed in order to describe the issues more precisely.

 1. In Introduction:

- The hypothesis that early inhibition of acute inflammation may lead to subacute or chronic state is commonly known, therefore more references are needed than only paper #7.
- Authors state that a blunted febrile response may be associated with increased mortality. In fact this issue is still under discussion. It is noteworthy that in some studies NSAIDs had no effect on the course of the disease or even had beneficial effects. Please, include this information in Introduction.
- Reference #18 concerns ferrets. Other reference that concerns humans should be provided.
- One of the most common mistakes in many articles about fever is treating hyperthermia as equal to fever. Unlike hyperthermia, fever is controlled elevation of body temperature due to the upward resetting of the hypothalamic thermostat. Therefore, instead of reference #19 about the effect of hyperthermia on isolated cells, please provide other paper about fever.

2. In Research question:

 Authors are going to investigate infectious fever. Which factor/s will be taken into account to differentiate infectious fever from non-infectious fever?

 3. In Methods/Search strategy:

 Please provide a list of key words that will be asked precisely. Adding key words such as Coxibs, NSAIDS, acetylsalicylic acid (ASA) should be considered.

 4. In Methods/ Study eligibility criteria:

 The exclusion criteria should be described more in detail. The research is targeted on the population who take antipyretics solely for fever. Taking NSAIDs due to its analgesic properties and for anti-plateled therapy should not be analysed.

 In my opinion after minor revision of this protocol this study will make an important contribution to the knowledge about geriatrics and hopefully will finally answer the intriguing question whether taking antipyretics can modify recovery of patients.

Is the study design appropriate for the research question?

Yes

Is the rationale for, and objectives of, the study clearly described?

Partly

Are sufficient details of the methods provided to allow replication by others?

Yes

Are the datasets clearly presented in a useable and accessible format?

Not applicable

Reviewer Expertise:

Fever, inflammation, immunity, antipyretics.

I confirm that I have read this submission and believe that I have an appropriate level of expertise to confirm that it is of an acceptable scientific standard.

# Response

**Published:** 2021-03-22

**Authors:** Seema Mahesh

**Affiliations:** Taylor's University, Malaysia

Thank you Dr Wrotek for your valuable suggestions. We will revise the paper accordingly and publish

 Thanking you

 Seema Mahesh

 Corresponding author
